# Nutritional Status Differs by Prescription Opioid Use among Women of Reproductive Age: NHANES 1999–2018

**DOI:** 10.3390/nu15081891

**Published:** 2023-04-14

**Authors:** Emily E. Hohman, Tammy E. Corr, Sarah Kawasaki, Jennifer S. Savage, Danielle Symons Downs

**Affiliations:** 1 Center for Childhood Obesity Research, Pennsylvania State University, University Park, PA 16802, USA; 2 Department of Pediatrics, Penn State College of Medicine, Hershey, PA 17033, USA; 3 Department of Psychiatry and Behavioral Health, Penn State College of Medicine, Hershey, PA 17033, USA; 4 Department of Nutritional Sciences, Pennsylvania State University, University Park, PA 16802, USA; 5 Department of Kinesiology, Pennsylvania State University, University Park, PA 16802, USA; 6 Department of Obstetrics and Gynecology, Penn State College of Medicine, Hershey, PA 17033, USA

**Keywords:** prescription opioids, preconception, women, nutritional status, biomarkers, NHANES

## Abstract

Prescription opioid use among pregnant women has increased in recent years. Prenatal exposure to opioids and poor nutrition can both negatively impact maternal–fetal outcomes. The objective of this study was to characterize the nutrition and health status of reproductive-age women taking prescription opioids, compared to women not taking opioids. Using NHANES 1999–2018 data, non-pregnant women aged 20–44 years were classified as taking a prescription opioid in the last 30 days (*n* = 404) or unexposed controls (*n* = 7234). Differences in anthropometric, cardiovascular, hematologic, and micronutrient status indicators between opioid-exposed and unexposed women were examined. Opioid-exposed women were older, had lower income and education, and were more likely to be non-Hispanic White, to smoke, and to have chronic health conditions compared to unexposed women. In unadjusted analyses, several nutrition and health markers were significantly different between opioid exposure groups. After controlling for covariates, women taking opioids had higher odds of Class II (OR = 1.6, 95% CI = 1.1–2.3) or III obesity (OR = 1.6, 95% CI = 1.1–2.5), and lower levels of serum folate, iron, and transferrin saturation. Reproductive-age women taking prescription opioids may be at risk for poorer nutritional and cardiometabolic health. Future research is needed to explore whether nutritional status impacts maternal–fetal outcomes for women exposed to opioids during pregnancy.

## 1. Introduction

Prescription opioid use is common among women of reproductive age. In 2008–2012, 27.7% of privately insured and 39.4% of Medicaid-enrolled women aged 15–44 years filled an opioid prescription [1]. Similarly, prescribing of opioid medications during pregnancy has increased over the last two decades [2], with an estimated 6 to 28% of pregnancies potentially exposed to prescription opioids [3,4,5,6]. There is also emerging evidence that childbirth in itself is a vulnerable opportunity for opioid exposure, as many clinicians recommend opioids for pain management after delivery [7]. Although additional research is needed, there is some evidence of associations between prenatal prescription opioid use and adverse maternal and neonatal outcomes, such as pre-eclampsia [8], hemorrhage [8], low birth weight [3], small for gestational age birth [6], large for gestational age birth [8], preterm birth [8], and birth defects [9,10]. Additionally, infants born to mothers taking prescription opioid medications are at risk for neonatal opioid withdrawal syndrome (NOWS). The risk of NOWS increases with longer-term maternal use of opioids, later gestational exposure, and the presence of other risk factors, such as smoking or concomitant use of prescription psychotropic medications [11,12]. Other factors that may exacerbate or mitigate the effect of prescription opioids on maternal and fetal outcomes, such as nutritional status, are not well understood. Emerging evidence suggests that maternal nutritional status may play a role in determining the severity of outcomes associated with other types of prenatal exposures, including alcohol [13] and stress [14], but whether this is the case for fetal opioid exposure is unknown.

Adequate maternal nutrition during pregnancy is crucial for proper fetal growth and development [15], optimal birth outcomes [16], and long-term offspring health [17]. Maternal nutrition-related health issues, such as obesity [18], diabetes [19], and hypertension [20], are also associated with increased risk for maternal and fetal complications. In particular, maternal nutritional status at the time of conception and in early pregnancy is particularly important for nutrients such as folate [21]. Nearly half of pregnancies in the US are unplanned [22], and thus, non-pregnant women of reproductive age represent a proxy population to examine nutritional status during peri-conception and early pregnancy.

Because both prescription drug and nutrient exposure can influence pregnancy and child health outcomes, the interaction between maternal prescription drug exposure and nutritional status on maternal and child health outcomes is of special interest. While interactions between maternal opioid use and nutritional status have not been examined in the published literature, there is some evidence to suggest that maternal nutritional status may moderate the effects of prenatal alcohol exposure [23,24,25]. Thus, it is important to understand whether women taking prescription opioids represent a group at increased risk for poor nutritional status. A few studies have identified increased risk of nutritional deficiencies, including folate, vitamin B12, and iron insufficiencies, in mid- to late pregnancy among women receiving medication for opioid use disorder [26,27], but whether the same is true for women taking any type of prescription opioid or in preconception/early pregnancy is unknown. This analysis characterizes nutritional and health status indicators among women of reproductive age in the National Health and Nutrition Examination Survey (NHANES) dataset who were taking prescription opioids and compares them with women not taking these drugs.

## 2. Materials and Methods

### 2.1. Data and Participants

NHANES is a series of nationally representative, cross-sectional surveys of non-institutionalized US residents [28]. The survey is conducted in two-year cycles and uses a complex, stratified, multistage probability cluster-sampling design. The NHANES examination protocol includes an interview including demographic, socioeconomic, dietary, and health-related questions, as well as physiological measurements and laboratory tests. For the purpose of this analysis, data were combined from 10 survey cycles spanning 1999–2018. The analytical sample included women of reproductive age. Though typically defined as 16–49 years, this analysis included only those between 20–44 years due to restrictions in the publicly available dataset (illicit drug use data is not available for participants <20 years, and pregnancy data is not available for participants >44 years). Women were excluded from analysis if they were pregnant at the time of the NHANES examination, or if they reported any heroin, cocaine, or methamphetamine use in the past year. Participants with incomplete covariate data were also excluded. Covariates included demographic and health history factors that were potentially related to prescription opioid use and/or health and nutritional status markers. The selected demographic covariates included age (years), race/ethnicity (Mexican American, other Hispanic, non-Hispanic white, non-Hispanic black, and other race/multiracial), education (less than high school, high school graduate/GED or equivalent, some college/associate’s degree, and college graduate or higher), marital status (married, living with partner, divorced/separated/widowed, and never married), family income to poverty threshold ratio (<1.30, 1.30–3.49, ≥3.50), employment (of any type, yes/no), and health insurance (any type, yes/no). Health history covariates included variables related to alcohol and tobacco use, chronic disease history, and reproductive history. Alcohol use was categorized into three levels using the National Institute on Alcohol Abuse and Alcoholism’s definition of low risk drinking [29]. Women who reported consuming no more than 3 drinks per day and no more than 7 drinks per week were classified as “low risk” drinkers, and those exceeding either of these thresholds were classified as “high risk” drinkers. Participants reporting no alcohol consumption in the past 12 months were classified as “non-drinkers”. Participants were classified as current smokers if they reported currently smoking cigarettes every day or on some days. Chronic disease history was assessed according to responses to a number of questions asking if a doctor or health professional had ever told the participant that they had specific medical conditions. For this analysis, we included conditions that were (a) potentially related to prescription opioid use and/or health and nutritional status indicators, (b) included in all eight survey cycles, and (c) relatively common in women of reproductive age. The six selected conditions were arthritis (any kind), asthma, cancer (any kind), chronic bronchitis, diabetes/borderline diabetes, and thyroid condition. The total number of previous pregnancies (regardless of pregnancy outcome) was included as a continuous variable. Finally, though body mass index was a health outcome of interest, it was also included as a covariate for all other outcomes due to its strong, established link to a variety of health and nutritional outcomes. The final analytical sample included 404 women who reported taking prescription opioids and 7234 unexposed controls, although the exact numbers included in the analysis varied by outcome due to variability in which measures were included in each NHANES cycle.

### 2.2. Prescription Opioid Use

Participants were first asked if they had taken any prescription medications in the past month. If yes, interviewers asked to see containers/bottles for all prescription medications and recorded the medication names from the containers. If containers were not available, participants were asked to verbally report the name of the medication. Participants were also asked how long they had been taking the medication. All reported drugs were converted to generic drug names and classified by therapeutic use using the 3-level Cerner Multum Lexicon Therapeutic Classification Scheme (Cerner Corporation, Kansas City, MO, USA). Participants were classified as taking a prescription opioid if they reported a medication with a drug or ingredient therapeutic category ID of level 1: 58 (central nervous system agents), level 2: 60 (analgesics), and level 3: 60 (narcotic analgesics) or 191 (narcotic analgesic combinations). Participants who completed the prescription medication portion of the survey but did not report taking opioid medications were classified as unexposed controls.

### 2.3. Nutrition and Health Status Measurements

In all survey cycles, anthropometric measurements, blood pressure, and blood sample collection were completed during a physical exam at a mobile examination center. Blood was collected by venipuncture, and fasting status was assessed at the time of the blood draw. Details on measurement protocols and laboratory measurements, as well as information on study design and ethical approval, can be found on the NHANES website [28]. The selection of available nutritional status markers varies by survey cycle; thus, the analytical sample size for each outcome variable differs, depending on how many survey cycles include each particular marker in their analytical protocol.

### 2.4. Analysis

Nutritional and health status outcomes were analyzed as both continuous variables and as categories using clinically relevant cut-off values. Body mass index was categorized using cutoffs of <18.5 kg/m^2^ for underweight, 18.5–24.9 for normal weight, 25.0–29.9 for overweight, 30.0–34.9 for class I obesity, 35.0–39.9 for class II obesity, and ≥40 for class III obesity [30]. The following thresholds were used to categorize participants as having an abnormal or suboptimal value: waist circumference, >88 cm [31]; blood pressure, systolic BP ≥ 120 mm Hg or diastolic BP ≥ 90 mm Hg [32]; serum HDL, <50 mg/dL [33]; fasting serum LDL, >100 mg/dL [33]; fasting serum triglycerides, ≥150 mg/dL [33]; fasting plasma glucose, ≥100 mg/dL [34]; hemoglobin A1C, ≥5.7% [34]; hemoglobin, <12 g/dL [35]; hematocrit, <36% [36]; red blood cell count, <4.2 × 10^6^ cells/µL [36]; mean corpuscular volume, <80 fL (low) or >100 fL (high) [36]; serum ferritin, <15 µg/L (low) or >150 µg/L (high) [37]; serum iron, <40 µg/dL [38]; serum transferrin receptor, >5.33 mg/L [39]; serum transferrin saturation, <15% [40]; serum TIBC, >460 µg/dL [36]; serum folate, <10 nmol/L [41]; serum vitamin B12, <203 pmol/L [41]; plasma homocysteine, >13 µmol/L [42]; serum PLP, <20 nmol/L [43]; serum retinol, ≤0.70 µmol/L [44]; serum vitamin C, <11.4 μmol/L [45]; and serum 25OH vitamin D, <30 nmol/L [46].

Data were analyzed using SAS 9.4 (SAS Institute, Cary, NC, USA). All analyses were performed using NHANES examination weights. Sample weights for analyses combining multiple survey cycles were calculated as described in the NHANES analytic guidelines [28]. Analyses were conducted using SAS survey procedures, which account for the sampling weights and complex survey design of NHANES. Continuous variables were analyzed using PROC SURVEYMEANS and PROC SURVEYREG, and categorical variables were analyzed using PROC SURVEYFREQ and PROC SURVEYLOGISTIC. For each outcome, the effect of prescription opioid use was assessed in 3 models: (1) an unadjusted model containing no covariates; (2) a model adjusted for survey cycle and demographic covariates (age, race/ethnicity, education, marital status, income poverty ratio, employment, and health insurance); and (3) a model adjusted for survey cycle, demographic covariates, and health history covariates (BMI [except where BMI was the outcome], alcohol use, smoking, history of arthritis, asthma, chronic bronchitis, cancer, and diabetes, as well as number of previous pregnancies). Two sets of subgroup analyses were also conducted: first, dividing the sample into younger (20–34 years) and older (35–44 years) age groups and second, dividing the sample into women without obesity (BMI < 30 kg/m^2^) and women with obesity (BMI ≥ 30 kg/m^2^). These subanalyses were conducted due to the higher risk of pregnancy complications among mothers with advanced age and obesity. For simplicity, only the results of the unadjusted and fully adjusted models are described for subgroup analyses.

## 3. Results

**Figure 1:**
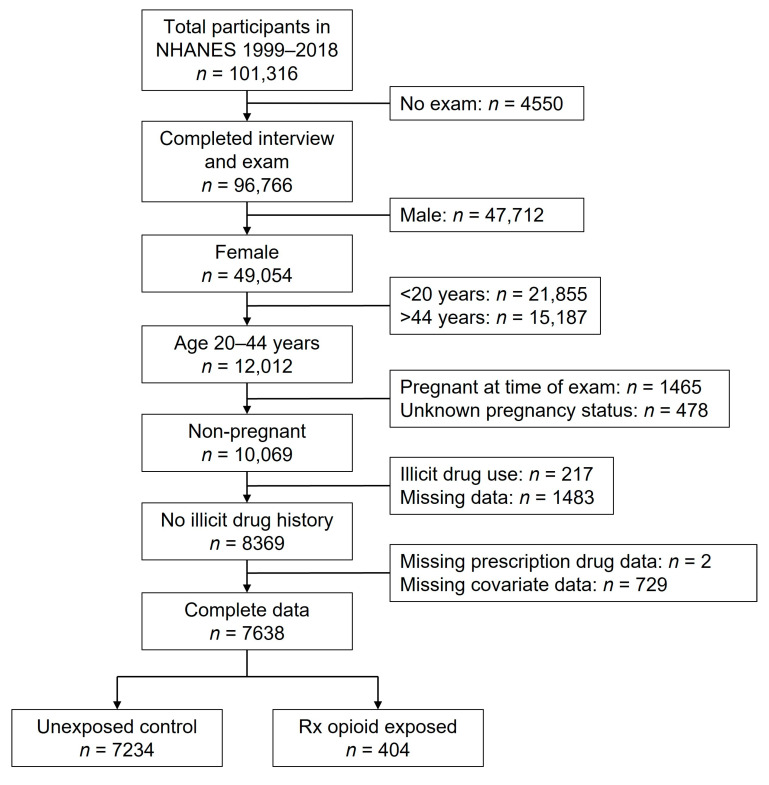
Participant flow diagram.

A total of 12,012 participants met the initial age and sex criteria (Figure 1). Of these, *n* = 4374 (36%) were excluded from analysis for the following reasons: pregnant at the time of the exam (*n* = 1465) or missing/unknown pregnancy status data (*n* = 478); use of heroin, cocaine, or methamphetamine in the previous year (*n* = 217) or missing illicit drug use data (*n* = 1483); missing prescription drug data (*n* = 2); or incomplete covariate data (*n* = 729, mostly due to missing income data, *n* = 545). Out of the remaining 7638 participants, 404 (5.3%) reported taking a prescription opioid medication in the previous month. The most commonly reported opioid was hydrocodone (48.3%), followed by oxycodone (17.8%), tramadol (12.1%), codeine (9.2%), propoxyphene (7.4%), and others (5.2%). The duration of use ranged from 1 day to 20 years, with a median of 61 days. A number of demographic and health history variables differed by prescription opioid use (Table 1). Compared to the unexposed group, women who reported taking prescription opioids were older, and greater percentages were non-Hispanic White, divorced/separated/widowed, had incomes <1.30 times the federal poverty line, and had health insurance. Those in the prescription opioid group were less likely to be college graduates, never married, and employed. Among health history variables, women in the prescription opioid-exposed group had a greater number of previous pregnancies, and greater percentages reported current cigarette smoking and history of arthritis, asthma, cancer, chronic bronchitis, diabetes/borderline diabetes, and thyroid condition. There was no significant difference between groups in level of alcohol use.

In unadjusted analyses, there were several health indicators that differed between the prescription opioid-exposed and unexposed control groups. Among cardiometabolic health parameters, women taking prescription opioids had a higher BMI, waist circumference, systolic blood pressure, fasting serum triglycerides, and hemoglobin A1C, and lower HDL cholesterol (Table 2). Except for systolic blood pressure and hemoglobin A1C, these differences persisted after adjustment for demographic factors; however, none were statistically significant after adjusting for health history covariates. There were no differences by prescription opioid use status in diastolic blood pressure, LDL cholesterol, fasting glucose, or fasting insulin in any models.

Among hematologic and iron status markers, women taking prescription opioids had lower serum iron and lower serum transferrin saturation (both unadjusted and adjusted for demographic and health history covariates, Table 2). No differences were seen in hemoglobin, hematocrit, serum ferritin, serum transferrin receptor, or serum total iron binding capacity. Three other micronutrient status indictors—serum folate, plasma homocysteine, and serum vitamin C—differed between the two groups: women taking prescription opioids had lower serum folate, higher plasma homocysteine, and lower vitamin C than unexposed women in the unadjusted models. Both serum folate and plasma homocysteine remained significantly different after adjusting for demographic characteristics, but only serum folate remained significant after adjustment for health history covariates. No difference in prescription opioid exposure was seen for serum vitamin B12, B6, retinol, or 25-hydroxyvitamin D levels.

Next, health and nutritional status markers were analyzed using clinically relevant cut offs to categorize status. For four markers (serum folate, serum vitamin B12, plasma homocysteine, and serum retinol), the prevalence of abnormal values in both groups was low (<3%), so these were not further analyzed. Across both groups, high rates of overweight/obesity and suboptimal cardiometabolic health markers were observed. In unadjusted analyses, women taking prescription opioids had greater odds of class II and III obesity, high waist circumference, elevated/hypertensive blood pressure, low HDL, elevated triglycerides, elevated fasting glucose, and meeting criteria for metabolic syndrome (Table 3). Of these, blood pressure, HDL, and fasting glucose were no longer statistically significant after adjustment for demographic characteristics, and waist circumference, triglycerides, and metabolic syndrome were not significant after adjusting for demographic and health history covariates. The risk of obesity among women taking prescription opioids remained significantly greater across all three models. Women taking prescription opioids had 1.6 times greater odds (CI: 1.1–2.3) of class II obesity (35–39.9 kg/m^2^), and 1.6 times greater odds (95% CI: 1.1–25) of class III (≥40 kg/m^2^) obesity, compared to unexposed control women.

Among hematologic and micronutrient status indicators, in unadjusted analyses, women taking prescription opioids had greater odds of high mean corpuscular volume, high serum ferritin, low serum transferrin saturation, and low serum vitamin B6 status (Table 3). Of these, serum ferritin was not significantly different after adjusting for demographic characteristics, and mean corpuscular volume and vitamin B6 status were not different after adjusting for demographic and heath history covariates. Low serum transferrin saturation was significantly different in all three models: women taking prescription opioids had 1.5 (95% CI: 1.1–2.2) times greater odds of having low transferrin saturation (<15%) than women not taking opioids. In addition, one relationship that was not significant in unadjusted analysis emerged after covariate adjustment; in the demographic-adjusted, but not the fully adjusted model, women taking opioids had higher odds of low 25-hydroxyvitamin D (OR = 1.6, 95% CI: 1.0–2.5).

### 3.1. Subgroup Analyses

#### 3.1.1. Age

In unadjusted continuous outcome analyses, women age 20–<35 years taking prescription opioids (*n* = 189) had higher BMI, waist circumference, fasting serum triglycerides, and plasma homocysteine, and lower serum iron, percent transferrin saturation, and folate levels, compared to unexposed women age 20–< 35 (*n* = 4172, Supplemental Table S1). In fully adjusted models, only BMI, ferritin, transferrin saturation, and folate levels remained significant. Two additional effects also emerged after covariate adjustment—younger women taking opioids had lower hemoglobin and hematocrit levels than those not taking opioids. In the unadjusted analysis of categorical outcomes, younger women taking prescription opioids had higher odds of class II and class III obesity and metabolic syndrome, high waist circumference, high triglycerides, low serum iron, high serum transferrin receptor, and low percent serum transferrin saturation (Supplemental Table S2). After adjustment for covariates, younger women taking opioids had higher odds of class III obesity (OR = 2.1, 95% CI: 1.2–3.7), high serum transferrin receptor (OR = 2.3, 95% CI: 1.2–4.30), and low percent transferrin saturation (OR = 2.6, 95% CI: 1.7–4.2) than unexposed younger women.

Among the older age group (35–45 years), in unadjusted continuous outcome analyses, women taking prescription opioids (*n* = 215) had higher BMI, waist circumference, diastolic blood pressure, fasting serum triglycerides, and hemoglobin, and lower HDL, serum folate, and vitamin C levels than unexposed older women (*n* = 3062, Supplemental Table S3). None of these were statistically significant in the fully adjusted models. In the unadjusted analysis of categorical outcomes, older women taking prescription opioids had higher odds of underweight, class II and III obesity, and metabolic syndrome, high waist circumference, high blood pressure, high triglycerides, high mean corpuscular volume, low vitamin B6, and low vitamin C levels, as well as lower odds of having low hemoglobin (Supplemental Table S4) After adjustment for covariates, older women taking prescription opioids had higher odds of class II obesity (OR = 1.8, 95% CI: 1.1–3.1) and high waist circumference (OR = 2.1, 95% CI: 1.1–4.0). Two additional effects also emerged in the fully adjusted model: older women taking prescription opioids had higher odds of low red blood cell count (OR = 1.7, 95% CI: 1.1–2.6) and low serum 25 hydroxyvitamin D (OR = 2.1, 95% CI: 1.0–4.1) than unexposed older women.

#### 3.1.2. Obesity

Among women without obesity (BMI < 30), in unadjusted continuous outcome analyses, women taking prescription opioids (*n* = 195) had higher waist circumference, fasting triglycerides, and mean corpuscular volume, and lower HDL, serum iron, percent transferrin saturation, and serum folate levels compared to women not taking prescription opioids (*n* = 4535, Supplemental Table S5). In fully adjusted models, only serum iron and percent transferrin saturation remained statistically significant. In the unadjusted analyses of categorical outcomes, women without obesity taking prescription opioids had higher odds of metabolic syndrome, high waist circumference, low HDL, high triglycerides, high mean corpuscular volume, high serum ferritin, and low serum transferrin saturation (Supplemental Table S6). After adjustment for covariates, women without obesity taking prescription opioids had higher odds of metabolic syndrome (OR = 3.4, 95% CI: 1.4–8.3), high triglycerides (OR = 2.6, 95% CI: 1.3–5.0), and low percent transferrin saturation (OR = 1.8, 95% CI: 1.1–3.0) than unexposed women without obesity.

Among women with obesity (BMI ≥ 30), in unadjusted continuous outcome analyses, those taking prescription opioids (*n* = 209) had higher BMI, waist circumference, fasting triglycerides, and lower vitamin B6 levels than those not taking prescription opioids (*n* = 2699, Supplemental Table S7). None of these associations were significant after adjusting for covariates. In the unadjusted analysis of categorical outcomes, women with obesity taking prescription opioids had higher odds of high triglycerides, low red blood cell count, low vitamin B6, and low vitamin C (Supplemental Table S8). After adjustment for covariates, women with obesity taking prescription opioids had higher odds of low red blood cell count (OR = 1.8, 95% CI: 1.1–2.8), and two additional significant effects emerged: higher odds of high transferrin receptor (OR = 2.2, 95% CI: 1.3–3.9), as well as lower odds of low HDL (OR = 0.6, 95% CI: 0.5–0.9).

## 4. Discussion

In this analysis of reproductive-age women, ages 20–44 years, we found that those who reported taking a prescription opioid within the last month were significantly more likely than those who had not taken prescription opioids to have suboptimal nutritional status and cardiometabolic health; however, many of these differences were attributable to demographic or other health history characteristics. Regardless of whether these differences in nutritional status are related to prescription opioid use per se or a result of other factors, these results suggest that women taking prescription opioids represent a group at increased risk for poor nutritional status during pregnancy, and thus possibly increased risk for adverse pregnancy outcomes.

Women taking prescription opioids were more likely than unexposed women to have BMIs that classified them as having class II or III obesity. This finding is consistent with another recent analysis of NHANES data showing increased risk of prescription opioid use with increasing BMI in middle-aged men and women [47], and an analysis of adults with back pain from the Medical Expenditure Panel Survey showing higher rates of opioid prescription use among those with obesity [48]. High pre-pregnancy BMI is well-established as a risk factor for adverse pregnancy outcomes, including gestational diabetes, pre-eclampsia, large for gestational age birth, preterm birth, cesarean section, and NICU admission [49,50,51]. Some of these complications, such as diabetes and preterm birth, have also been associated with maternal opioid exposure during pregnancy [52]. Further research is needed to determine what role maternal weight status plays in the risk of adverse pregnancy outcomes among opioid exposed women.

Women taking prescription opioids had greater odds of having several adverse cardiometabolic measures, although these effects were not apparent after controlling for health covariates, including BMI and smoking. Other studies have identified increased risk of hypertension, dyslipidemia, and diabetes among adults taking prescription opioids [53,54]. Pre-pregnancy dyslipidemia has been associated with increased risk for preeclampsia, gestational diabetes, and pre-term birth [55,56], and women with metabolic syndrome had greater risk for pregnancy complications [57]. Thus, poor cardiometabolic health among women taking prescription opioids may exacerbate the risk for adverse pregnancy outcomes.

Other published work has reported an association between long-term prescription opioid use and increased risk of iron deficiency anemia [58]. In regards to associations between prescription opioid use and hematological and iron status markers, we observed mixed findings. While there was no difference between groups in regards to hemoglobin, hematocrit, or ferritin levels, women taking prescription opioids had lower serum iron and percent transferrin saturation. Iron transport markers, such as transferrin saturation, are impacted earlier in the process of iron depletion than is functional iron (e.g., hemoglobin) [35], suggesting that non-pregnant women taking prescription opioids may have increased risk of mild iron deficiency. This could translate to increased risk for iron deficiency anemia in pregnancy, when iron stores are further taxed by expansion of maternal blood volume and the developing fetus [35]. Iron deficiency during pregnancy is a risk factor for preterm birth, small for gestational age birth, and maternal perinatal bleeding, and may also have longer-term impacts on infant cognitive, motor, and social-emotional development [59].

Although the percentage of women with inadequate folate status (<10 nmol/L) was low in both groups, women taking prescription opioids had a lower mean serum folate status than unexposed women. A similar pattern was observed with elevated homocysteine, an indicator of folate or vitamin B12 deficiency [60]; however, there was no difference in serum vitamin B12 by prescription opioid status. In the US, the prevalence of folate deficiency and related consequences in pregnancy (e.g., neural tube defects) have decreased substantially due to food fortification and periconceptional supplementation with folate [21]. While few women taking prescription opioids fell below the threshold for serum folate deficiency, the pattern of differences observed suggests that women taking opioids may have a somewhat increased risk for folate inadequacy and should follow current public health guidance related to folate supplementation in the periconceptional period. Previous work has similarly found lower serum folate and higher homocysteine levels in pregnant women receiving methadone therapy compared to controls [26].

In the subgroup analyses by age, there were both similarities and differences between younger and older women in the association between prescription opioid use and outcomes. Prescription opioid use was associated with greater risk of severe obesity in both the younger and older groups. However, associations with iron and folate status markers were observed only among the younger age group. Other examinations of NHANES data have found that pregnant women aged 20–34 years had greater prevalence of iron deficiency than did pregnant women aged 35–49 years [61]. Younger women taking prescription opioids may be at further risk for developing iron deficiency in pregnancy. In a Norwegian sample of male and female patients receiving medication for opioid use disorder, younger individuals had lower serum folate concentrations than older individuals [62]. Thus, low folate may be of particular concern in early pregnancy for younger women taking opioids.

In the subanalyses by obesity status, relationships between opioid use and adverse health status appeared to be stronger among women without obesity. In this group, women who were taking prescription opioids had a greater risk of metabolic syndrome, elevated triglycerides, and poor iron status than those not taking opioids. The lack of difference by opioid use among women with obesity may be due to the fact that the prevalence of these nutritional problems is already elevated among women with obesity. While obesity is commonly associated with poor metabolic health, metabolic syndrome does occur in lean individuals and has been linked to lifestyle factors such as poor diet [63] and smoking [64]. Opioid use may be another risk factor for poor metabolic health in women without obesity.

A limitation of this work is that not all outcome variables were available for all survey years. Thus, there are some outcomes for which the analytical sample size is considerably smaller than others. Moreover, although NHANES is a representative sample of the United States, the small number of individuals included from some racial and ethnic minority groups may limit the applicability of these results across a broad population.

## 5. Conclusions

In summary, non-pregnant reproductive-age women taking prescription opioids have a greater prevalence of some indicators of adverse cardiometabolic health and nutritional status compared to women not taking opioids. While many of these risks seem to be attributable to other health and demographic factors associated with prescription opioid use, poor health and nutritional status among women taking opioids has the potential to further increase risks for adverse outcomes if women taking opioids become pregnant. Preconception and pregnant women who take opioids should be evaluated for potential nutritional risk and follow medical guidance to optimize nutritional status during these important life stages. Future research should examine the extent to which maternal nutritional status moderates the effects of prenatal opioid exposure on maternal and infant health outcomes.

## Figures and Tables

**Table 1:** Demographic and health history characteristics of reproductive-age women by prescription opioid use status, NHANES 1999–2018.

Variable	Prescription Opioid Exposed (n = 404)	Unexposed (n = 7234)	*p*-Value
Mean (SE)			
Age (years)	33.9 (0.4)	32.4 (0.1)	0.0001
Number of previous pregnancies	2.7 (0.1)	2.0 (0.04)	<0.0001
Percent (SE)			
Race/ethnicity			0.0008
Mexican American	8.4 (1.2)	9.7 (0.7)	
Other Hispanic	3.0 (0.9)	6.6 (0.5)	
Non-Hispanic White	72.5 (2.6)	63.6 (1.2)	
Non-Hispanic Black	12.0 (1.7)	12.7 (0.7)	
Other/Multiracial	4.2 (1.1)	7.3 (0.4)	
Education			<0.0001
Less than high school	17.0 (2.0)	12.0 (0.5)	
High school grad or equivalent	25.2 (2.4)	19.9 (0.7)	
Some college/assoc. degree	39.1 (2.8)	36.1 (0.9)	
College grad or higher	18.7 (2.8)	32.1 (1.1)	
Marital status			<0.0001
Married	47.6 (3.0)	49.4 (0.9)	
Living with partner	14.8 (1.9)	11.2 (0.5)	
Divorced/separated/widowed	19.0 (2.1)	11.2 (0.5)	
Never married	18.6 (2.2)	28.2 (0.9)	
Income to poverty ratio			0.0003
<1.30	36.0 (2.9)	25.6 (0.8)	
1.30–3.49	36.2 (3.2)	36.8 (0.8)	
≥3.50	27.9 (2.9)	37.6 (1.0)	
Employed	56.9 (2.7)	73.5 (0.8)	<0.0001
Health insurance (any type)	86.3 (1.9)	79.3 (0.7)	0.002
Alcohol use			0.07
Non-drinker	28.9 (3.3)	22.4 (0.8)	
Low risk	56.3 (3.6)	61.1 (0.9)	
High risk	14.9 (1.9)	16.5 (0.6)	
Current cigarette smoking	39.5 (2.8)	21.2 (0.7)	<0.0001
Ever diagnosed with			
Arthritis (any type)	33.2 (2.5)	8.7 (0.5)	<0.0001
Asthma	31.9 (3.0)	16.4 (0.6)	<0.0001
Cancer (any type)	12.2 (2.0)	3.6 (0.3)	<0.0001
Chronic bronchitis	17.7 (2.2)	5.0 (0.4)	<0.0001
Diabetes or borderline diabetes	7.1 (1.2)	3.6 (0.3)	0.0001
Thyroid condition	12.7 (1.9)	7.9 (0.4)	0.002

**Table 2:** Cardiovascular, metabolic, hematologic, and micronutrient status measures in reproductive age women by prescription opioid status, NHANES 1999–2018.

Measure	Rx Opioid Exposed			Unexposed Control			Model 1 ^a^	Model 2 ^b^	Model 3 ^c^
	*n*	Mean	SE	*n*	Mean	SE	*p*	*p*	*p*
BMI (kg/m^2^)	404	30.6	0.5	7234	28.3	0.1	<0.0001	0.0005	0.05
Waist circumference (cm)	397	98.4	0.2	7137	92.4	0.3	<0.0001	0.0008	0.70
Systolic BP (mm Hg)	393	113.3	0.7	7050	111.7	0.2	0.04	0.25	0.67
Diastolic BP (mm Hg)	393	70.1	0.6	7050	69.0	0.2	0.06	0.51	0.85
HDL (mg/dL)	388	53.2	0.9	6956	56.4	0.3	0.0004	0.02	0.66
Fasting serum LDL cholesterol (mg/dL)	176	112.3	2.9	3096	106.6	0.7	0.06	0.51	0.87
Fasting serum triglycerides (mg/dL)	178	132.8	8.4	3114	100.0	1.9	0.0002	0.01	0.14
Fasting glucose (mg/dL)	180	07.2	1.4	3141	95.6	0.4	0.30	0.57	0.75
Hemoglobin A1C (%)	392	5.34	0.03	7000	5.27	0.01	0.049	0.23	0.69
Hemoglobin (g/dL)	393	13.46	0.07	7016	13.40	0.02	0.36	0.80	0.30
Hematocrit (%)	393	39.6	0.2	7016	39.5	0.1	0.60	0.78	0.28
RBC count (million cells/µL)	393	4.48	0.02	7016	4.48	0.01	0.87	0.99	0.16
Mean cell volume (fL)	393	88.8	0.3	7016	88.4	0.1	0.24	0.44	0.32
Serum ferritin (µg/L)	304	59.7	3.7	5442	53.6	0.8	0.11	0.22	0.39
Serum iron (µg/dL)	174	72.3	2.6	3065	82.7	1.0	0.0003	0.003	0.009
Serum transferrin receptor (mg/L)	239	3.75	0.18	4250	3.52	0.04	0.22	0.26	0.23
Serum transferrin saturation (%)	174	20.4	0.8	3060	23.4	0.3	0.0009	0.006	0.01
Serum total iron binding capacity (µg/dL)	174	365.8	6.8	3060	364.5	1.7	0.84	0.95	0.77
Serum folate (nmol/L)	390	36.5	1.1	6955	41.3	0.5	<0.0001	0.0005	0.02
Serum vitamin B12 (pmol/L)	233	374.8	11.4	3861	407.4	24.0	0.23	0.26	0.60
Plasma homocysteine (µmol/L)	150	7.68	0.29	2377	6.93	0.06	0.01	0.048	0.06
Serum PLP (vitamin B6) (nmol/L)	144	59.3	8.5	2220	63.6	2.6	0.62	0.80	0.91
Serum retinol (µmol/L)	149	1.92	0.07	2354	1.84	0.01	0.20	0.40	0.24
Serum vitamin C (μmol/L)	110	45.4	2.9	1894	53.0	1.1	0.01	0.07	0.63
Serum 25OH vitamin D (nmol/L)	304	64.7	2.2	4925	64.7	0.7	0.98	0.58	0.67

^a^ Unadjusted; ^b^ adjusted for survey cycle, age, race/ethnicity, education, marital status, income poverty ratio, employment, and health insurance; ^c^ adjusted for model 2 covariates and BMI (except where BMI is the outcome), alcohol use, smoking, history of arthritis, asthma, chronic bronchitis, cancer, diabetes, and number of previous pregnancies.

**Table 3:** Health and nutrition status using clinically relevant cutoffs among women of reproductive-age by prescription opioid use status.

Measure	Cutoff	Rx Opioid		Control		Model 1 ^a^	Model 2 ^b^	Model 3 ^c^
		%	SE	%	SE	OR (CI)	OR (CI)	OR (CI)
Body mass index	Underweight (<18.5 kg/m^2^)	3.8	1.4	2.9	0.2	1.8 (0.8–4.0)	1.7 (0.8–3.7)	1.6 (0.7–3.5)
	Overweight (25–29.9 kg/m^2^)	22.9	2.8	24.4	0.6	1.3 (0.9–1.9)	1.2 (0.8–1.8)	1.2 (0.8–1.7)
	Obese I (30–34.9 kg/m^2^)	16.5	2.1	16.6	0.5	1.4 (0.9–2.0)	1.2 (0.8–1.8)	1.0 (0.7–1.5)
	Obese II (35–39.9 kg/m^2^)	15.6	1.9	9.4	0.4	2.3 (1.7–3.3)	2.0 (1.4–3.2)	1.6 (1.1–2.3)
	Obese III (≥40.0 kg/m^2^)	14.2	1.9	8.2	0.4	2.4 (1.7–3.5)	2.2 (1.5–3.2)	1.6 (1.1–2.5)
Waist circumference	High (>88 cm)	66.2	2.9	52.7	0.8	1.8 (1.4–2.3)	1.5 (1.2–2.0)	1.2 (0.8–1.8)
Blood pressure	High (≥120/80 mm Hg)	29.8	2.2	24.6	0.6	1.3 (1.1–1.6)	1.2 (0.9–1.5)	1.0 (0.8–1.3)
Serum HDL cholesterol	Low (<50 mg/dL)	43.8	3.1	35.8	0.8	1.4 (1.1–1.8)	1.2 (0.9–1.5)	0.8 (0.6–1.1)
Fasting LDL cholesterol	High (>100 mg/dL)	59.0	4.4	54.2	1.2	1.2 (0.8–1.8)	1.0 (0.7–1.5)	0.9 (0.6–1.3)
Fasting serum triglycerides	High (≥150 mg/dL)	30.1	3.9	14.4	0.8	2.6 (1.8–3.7)	2.0 (1.4–2.9)	1.5 (1.0–2.4)
Fasting plasma glucose	High (≥100 mg/dL)	29.4	3.9	21.4	0.9	1.5 (1.0–2.2)	1.5 (1.0–2.2)	1.2 (0.8–1.9)
Hemoglobin A1C	High (≥5.7%)	14.0	1.9	10.8	0.5	1.3 (1.0–1.9)	1.3 (0.9–1.8)	0.9 (0.6–1.4)
Metabolic syndrome	Meets 3 or more criteria	35.5	4.2	18.0	0.7	2.5 (1.7–3.7)	2.1 (1.4–3.2)	1.6 (1.0–2.6)
Hemoglobin	Low (<12 g/dL)	7.1	1.4	8.6	0.4	0.8 (0.6–1.2)	0.8 (0.5–1.2)	0.9 (0.5–1.3)
Hematocrit	Low (<36%)	11.0	1.7	10.7	0.5	1.0 (0.7–1.5)	1.0 (0.7–1.5)	1.1 (0.8–1.7)
RBC count	Low (<4.2 × 10^6^ cells/µL)	22.4	2.4	19.4	0.7	1.2 (0.9–1.6)	1.1 (0.9–1.5)	1.3 (1.0–1.7)
Mean corpuscular volume	Low (<80 fL)	7.4	1.3	7.2	0.3	1.1 (0.7–1.6)	1.1 (0.7–1.6)	1.1 (0.7–1.5)
	High (>100 fL)	2.8	1.0	0.6	0.1	4.7 (2.0–11.0)	2.5 (1.1–5.7)	2.4 (1.0–5.8)
Serum ferritin	Low (<15 µg/L)	12.6	1.2	15.3	0.5	0.8 (0.6–1.2)	0.8 (0.6–1.2)	0.9 (0.6–1.4)
	High (>150 µg/L)	8.5	2.1	4.7	0.4	1.8 (1.1–3.2)	1.6 (0.9–2.8)	1.4 (0.8–2.5)
Serum iron	Low (<40 µg/dL)	15.6	3.2	12.3	0.7	1.5 (0.8–2.6)	1.2 (0.7–2.1)	1.1 (0.7–2.0)
Serum transferrin receptor	High (>5.33 mg/L)	11.7	2.0	8.4	0.5	1.5 (1.0–2.2)	1.4 (0.9–2.2)	1.5 (1.0–2.3)
Serum transferrin saturation	Low (<15%)	35.3	3.6	25.2	1.0	1.6 (1.2–2.2)	1.6 (1.1–2.3)	1.5 (1.1–2.2)
Serum TIBC	High (>460 µg/dL)	9.1	2.4	7.1	0.6	1.3 (0.7–2.3)	1.3 (0.7–2.4)	1.4 (0.7–2.5)
Serum PLP (vitamin B6)	Low (<20 µmol/L)	23.1	3.7	13.5	1.0	1.9 (1.3–2.9)	1.6 (1.0–2.5)	1.2 (0.7–1.9)
Serum vitamin C	Low (<11.4 μmol/L)	10.8	3.4	6.3	0.7	1.8 (0.9–3.4)	1.3 (0.6–2.6)	0.8 (0.4–1.6)
Serum 25OH Vitamin D	Low (<30 nmol/L)	9.7	1.9	7.7	0.6	1.3 (0.9–1.9)	1.6 (1.0–2.5)	1.5 (1.0–2.4)

^a^ Unadjusted; ^b^ adjusted for survey cycle, age, race/ethnicity, education, marital status, income poverty ratio, employment, and health insurance; ^c^ adjusted for model 2 covariates and BMI (except where BMI category is the outcome), alcohol use, smoking, history of arthritis, asthma, chronic bronchitis, cancer, diabetes, thyroid conditions, and number of previous pregnancies.

## Data Availability

The data were obtained from the National Health and Nutrition Examination Survey and are freely available at https://www.cdc.gov/nchs/nhanes/, accessed on 25 June 2021.

## Supplementary Materials

The following supporting information can be downloaded at: https://www.mdpi.com/article/10.3390/nu15081891/s1, Table S1: Cardiovascular, metabolic, hematologic, and micronutrient status measures among women aged 20–34 years by prescription opioid status, NHANES 1999–2018; Table S2: Health and nutrition status using clinically relevant cutoffs among women aged 20–34 years by prescription opioid use status; Table S3: Cardiovascular, metabolic, hematologic, and micronutrient status measures among women aged 35–44 years by prescription opioid status, NHANES 1999–2018; Table S4: Health and nutrition status using clinically relevant cutoffs among women aged 35–44 years by prescription opioid use status; Table S5: Cardiovascular, metabolic, hematologic, and micronutrient status measures among women with BMI < 30 by prescription opioid status, NHANES 1999–2018; Table S6: Health and nutrition status using clinically relevant cutoffs among women with BMI < 30 by prescription opioid use status; Table S7: Cardiovascular, metabolic, hematologic, and micronutrient status measures among women with BMI ≥ 30 by prescription opioid status, NHANES 1999–2018; Table S8: Health and nutrition status using clinically relevant cutoffs among women with BMI ≥ 30 by prescription opioid use status.
