# Supplementary material for: Nutritional Status Differs by Prescription Opioid Use among Women of Reproductive Age: NHANES 1999–2018
Source: Nutrients. 2023 Apr 14;15(8):1891. doi: 10.3390/nu15081891 (PMC10144164; doi:10.3390/nu15081891)
Supplement: Supplementary file 1 [file nutrients-15-01891-s001.zip › nutrients-2344169-supplementary.pdf]

Table S1. Cardiovascular, metabolic, hematologic, and micronutrient status measures among women age 20–34 years by prescription opioid status, NHANES 1999–2018.

Measure	Rx Opioid Users			Unexposed Control			Model 1 [a]	Model 2 [b]	Model 3 [c]
	n	Mean	SE	*n*	Mean	SE	*p*	*p*	*p*
BMI (kg/m^2)	189	30.1	0.7	4172	27.7	0.2	**0.001**	**0.002**	**0.02**
Waist circumference (cm)	186	95.9	1.6	4119	91.8	0.4	**0.001**	**0.003**	0.56
Systolic BP (mm Hg)	184	110.2	0.8	4074	110.0	0.2	0.77	0.99	0.29
Diastolic BP (mm Hg)	184	66.3	0.7	4074	67.0	0.2	0.38	0.23	0.08
HDL (mg/dL)	185	53.7	1.2	3993	55.9	0.3	0.21	0.21	0.48
Fasting serum LDL cholesterol (mg/dL)	77	103.7	3.9	1806	102.0	0.9	0.68	0.78	0.43
Fasting serum triglycerides (mg/dL)	77	115.9	8.8	1812	92.9	1.7	**0.01**	**0.03**	0.18
Fasting glucose (mg/dL)	78	92.2	1.4	1825	93.6	0.4	0.32	0.28	0.20
Hemoglobin A1C (%)	187	5.18	0.03	4018	5.19	0.01	0.70	0.72	0.06
Hemoglobin (g/dL)	187	13.32	0.09	4028	13.44	0.03	0.18	0.11	**0.03**
Hematocrit (%)	187	39.3	0.2	4028	39.6	0.1	0.14	0.13	**0.03**
RBC count (million cells/µL)	187	4.47	0.03	4028	4.50	0.01	0.35	0.41	0.08
Mean cell volume (fL)	187	88.2	0.5	4028	88.2	0.1	0.96	0.79	0.99
Serum ferritin (µg/L)	145	49.1	4.2	3092	50.0	1.0	0.83	0.76	0.42
Serum iron (µg/dL)	81	67.3	4.2	1741	84.0	1.3	**0.0002**	**0.0003**	**0.002**
Serum transferrin receptor (mg/L)	115	3.75	0.15	2437	3.44	0.04	0.06	0.07	0.08
Serum transferrin saturation (%)	81	18.4	1.2	1739	23.5	0.4	**0.0001**	**0.0001**	**0.0005**
Serum total iron binding capacity (µg/dL)	81	374.0	8.3	1739	366.9	1.9	0.40	0.43	0.41
Serum folate (nmol/L)	187	35.9	1.4	3990	41.3	0.8	**0.0009**	**0.0005**	**0.02**
Serum vitamin B12 (pmol/L)	111	382.3	15.1	2230	411.3	41.8	0.52	0.62	0.82
Plasma homocysteine (µmol/L)	71	7.61	0.45	1348	6.64	0.07	**0.04**	0.06	0.08
Serum PLP (vitamin B6) (nmol/L)	67	67.7	16.1	1277	60.6	3.1	0.66	0.65	0.49
Serum retinol (µmol/L)	70	1.89	0.12	1331	1.85	0.02	0.68	0.75	0.72
Serum vitamin C (µmol/L)	51	52.0	4.3	1095	53.5	1.4	0.74	0.88	0.64
Serum 25OH vitamin D (nmol/L)	144	66.0	3.3	2856	64.8	0.8	0.71	0.79	0

[a] Unadjusted, [b] Adjusted for survey cycle, age, race/ethnicity, education, marital status, income poverty ratio, employment, and health insurance, [c] Adjusted for model 2 covariates and BMI (except where BMI is the outcome), alcohol use, smoking, history of arthritis, asthma, chronic bronchitis, cancer, and diabetes, and number of previous pregnancies.

Table S2. Health and nutrition status using defined using clinically relevant cutoffs among women age 20–34 years by prescription opioid use status.

Measure	Cutoff	Rx Opioid		Control		Model 1 [a]	Model 2 [b]	Model 3 [c]
		%	SE	%	SE	OR (CI)	OR (CI)	OR (CI)
Body mass index	Underweight (<18.5 kg/m^2)	3.7	1.9	3.8	0.4	1.3 (0.4-4.0)	1.2 (0.4-3.6)	1.1 (0.4-2.8)
	Overweight (25–29.9 kg/m^2)	24.3	3.9	23.8	0.8	1.4 (0.9-2.2)	1.3 (0.8-2.0)	1.3 (0.8-2.0)
	Obese I (30–34.9 kg/m^2)	13.0	2.7	14.8	0.7	1.2 (0.7-2.0)	1.1 (0.6-2.0)	1.0 (0.5-1.7)
	Obese II (35–39.9 kg/m^2)	12.9	2.7	9.0	0.6	**1.9 (1.1-3.3)**	1.7 (1.0-3.0)	1.5 (0.8-2.7)
	Obese III (≥40.0 kg/m^2)	15.1	2.8	7.4	0.5	**2.7 (1.7-4.4)**	**2.7 (1.7-4.5)**	**2.1 (1.2-3.7)**
Waist circumference	High (>88 cm)	57.9	4.1	47.6	1.1	**1.5 (1.1-2.1)**	**1.4 (1.0-2.0)**	0.6 (0.3-1.5)
Blood pressure	High (≥120/80 mm Hg)	15.8	3.1	17.7	0.7	0.9 (0.6-1.4)	0.8 (0.5-1.3)	0.6 (0.4-1.0)
Serum HDL cholesterol	Low (<50 mg/dL)	43.5	4.0	35.4	1.0	**1.4 (1.0-1.9)**	1.3 (1.0-1.8)	0.9 (0.6-1.3)
Fasting LDL cholesterol	High (>100 mg/dL)	46.2	6.9	48.2	1.5	0.9 (0.5-1.6)	0.8 (0.5-1.4)	0.7 (0.4-1.2)
Fasting serum triglycerides	High (≥150 mg/dL)	25.8	6.4	12.0	1.0	**2.6 (1.4-4.9)**	**2.3 (1.2-4.2)**	1.8 (0.9-3.7)
Fasting plasma glucose	High (≥100 mg/dL)	18.9	5.3	17.2	1.1	1.1 (0.6-2.2)	1.0 (0.5-2.2)	0.8 (0.3-1.9)
Hemoglobin A1C	High (≥5.7%)	6.5	1.8	7.0	0.4	0.9 (0.5-1.7)	1.0 (0.5-1.8)	0.6 (0.3-1.1)
Metabolic syndrome	Meets 3 or more criteria	26.3	6.0	14.2	0.9	**2.2 (1.2-4.0)**	2.0 (1.0-3.8)	1.3 (0.6-3.2)
Hemoglobin	Low (<12 g/dL)	7.5	2.0	6.9	0.5	1.1 (0.6-1.9)	1.2 (0.7-2.1)	1.3 (0.7-2.2)
Hematocrit	Low (<36%)	11.8	2.7	9.2	0.6	1.3 (0.8-2.2)	1.4 (0.8-2.4)	1.5 (0.9-2.5)
RBC count	Low (<4.2 × 10^6 cells/µL)	18.5	3.1	18.5	0.9	1.0 (0.7-1.5)	1.0 (0.6-1.5)	1.0 (0.6-1.6)
Mean Cell Volume	Low (<80 fL)	7.8	1.7	6.5	0.4	1.2 (0.8-2.1)	1.3 (0.8-2.3)	1.1 (0.7-1.9)
	High (>100 fL)	1.6	1.0	0.4	0.1	3.9 (1.0-16.1)	**4.6 (1.6-13.2)**	3.6 (0.6-22.6)
Serum ferritin	Low (<15 µg/L)	12.7	3.3	13.7	0.7	1.0 (0.5-1.7)	1.0 (0.6-1.7)	1.0 (0.6-1.6)
	High (>150 µg/L)	6.2	2.7	3.1	0.3	2.0 (0.8-5.1)	2.1 (0.9-5.0)	1.9 (0.7-5.3)
Serum iron	Low (<40 µg/dL)	20.4	5.6	11.0	1.0	**2.6 (1.1-5.7)**	2.1 (1.0-4.5)	1.9 (0.9-4.1)
Serum transferrin receptor	High (>5.33 mg/L)	15.6	3.8	7.3	0.7	**2.3 (1.3-4.3)**	**2.4 (1.2-4.8)**	**2.3 (1.2-4.3)**
Serum transferrin saturation	Low (<15%)	46.3	5.2	24.0	1.3	**2.7 (1.8-4.3)**	**2.9 (1.8-4.6)**	**2.6 (1.7-4.2)**
Serum TIBC	High (>460 µg/dL)	11.2	3.8	7.4	0.8	1.6 (0.7-3.5)	1.6 (0.7-3.7)	1.6 (0.7-3.6)
Serum PLP (vitamin B6)	Low (<20 µmol/L)	20.7	5.4	13.1	1.2	1.7 (0.9-3.4)	1.7 (0.8-3.4)	1.2 (0.6-2.5)
Serum vitamin C	Low (<11.4 µmol/L)	2.3	2.3	5.7	1.1	0.4 (0.1-3.0)	0.3 (0.03-3.1)	0.2 (0.02-2.3)
Serum 25OH Vitamin D	Low (<30 nmol/L)	8.1	2.1	7.9	0.6	1.0 (0.6-1.9)	1.3 (0.7-2.5)	1.1 (0.6-2.1)

[a] Unadjusted, [b] Adjusted for survey cycle, age, race/ethnicity, education, marital status, income poverty ratio, employment, and health insurance, [c] Adjusted for model 2 covariates and BMI (except where BMI category is the outcome), alcohol use, smoking, history of arthritis, asthma, chronic bronchitis, cancer, diabetes, and thyroid conditions, and number of previous pregnancies.

Table S3. Cardiovascular, metabolic, hematologic, and micronutrient status measures among women age 35–44 years by prescription opioid status, NHANES 1999–2018.

	**Rx Opioid Users**			**Unexposed Control**			**Model 1 [a]**	**Model 2 [b]**	**Model 3 [c]**
**Measure**	*n*	**Mean**	**SE**	*n*	**Mean**	**SE**	*p*	*p*	*p*
BMI (kg/m^2)	215	31.0	0.6	3062	29.1	0.2	**0.005**	**0.04**	0.41
Waist circumference (cm)	211	100.6	1.6	3018	94.6	0.4	**0.0005**	**0.008**	0.33
Systolic BP (mm Hg)	209	116.0	1.2	2976	114.1	0.3	0.11	0.21	0.36
Diastolic BP (mm Hg)	209	73.5	0.8	2976	71.7	0.2	**0.03**	0.07	0.12
HDL (mg/dL)	203	52.7	1.2	2963	57.0	0.4	**0.0006**	**0.03**	0.86
Fasting serum LDL cholesterol (mg/dL)	99	118.4	4.0	1290	113.0	1.1	0.19	0.26	0.82
Fasting serum triglycerides (mg/dL)	101	144.7	12.7	1302	109.9	4.1	**0.01**	0.19	0.79
Fasting glucose (mg/dL)	102	100.7	2.0	1316	98.3	0.8	0.27	0.32	0.96
Hemoglobin A1C (%)	205	5.49	0.06	2982	5.38	0.01	0.07	0.18	0.44
Hemoglobin (g/dL)	206	13.60	0.09	2988	13.36	0.03	**0.01**	0.09	0.71
Hematocrit (%)	206	39.9	0.2	2988	39.4	0.1	0.05	0.12	0.86
RBC count (million cells/µL)	206	4.48	0.03	2988	4.46	0.01	0.35	0.55	0.43
Mean cell volume (fL)	206	89.3	0.5	2988	88.5	0.2	0.09	0.14	0.10
Serum ferritin (µg/L)	159	69.7	6.0	2350	58.0	1.5	0.06	0.12	0.48
Serum iron (µg/dL)	93	76.9	3.0	1324	81.2	1.4	0.20	0.60	0.74
Serum transferrin receptor (mg/L)	124	3.75	0.33	1813	3.62	0.05	0.70	0.66	0.55
Serum transferrin saturation (%)	93	22.1	1.1	1321	23.3	0.4	0.30	0.83	0.93
Serum total iron binding capacity (µg/dL)	93	358.5	8.9	1321	361.5	2.4	0.74	0.46	0.60
Serum folate (nmol/L)	203	37.2	1.7	2965	41.4	0.5	**0.01**	0.17	0.53
Serum vitamin B12 (pmol/L)	122	368.2	16.7	1631	402.3	9.8	0.08	0.13	0.20
Plasma homocysteine (µmol/L)	79	7.75	0.31	1029	7.28	0.11	0.12	0.76	0.92
Serum PLP (vitamin B6) (nmol/L)	77	52.3	10.6	943	67.4	4.2	0.21	0.51	0.84
Serum retinol (µmol/L)	79	1.95	0.08	1023	1.83	0.02	0.15	0.43	0.38
Serum vitamin C (µmol/L)	59	39.3	4.5	799	52.3	1.3	**0.008**	0.18	0.52
Serum 25OH vitamin D (nmol/L)	160	63.5	2.6	2069	64.7	0.9	0.66	0.38	0.80

[a] Unadjusted, [b] Adjusted for survey cycle, age, race/ethnicity, education, marital status, income poverty ratio, employment, and health insurance, [c] Adjusted for model 2 covariates and BMI (except where BMI is the outcome), alcohol use, smoking, history of arthritis, asthma, chronic bronchitis, cancer, and diabetes, and number of previous pregnancies.

Table S4. Health and nutrition status using defined using clinically relevant cutoffs among women age 35–44 years by prescription opioid use status.

		**Rx Opioid**		**Control**		**Model 1** [a]	**Model 2** [b]	**Model 3** [c]
**Measure**	**Cutoff**	**%**	**SE**	**%**	**SE**	**OR (CI)**	**OR (CI)**	**OR (CI)**
Body mass index	Underweight (<18.5 kg/m^2)	3.8	1.8	1.7	0.3	**3.3 (1.2-8.9)**	2.2 (0.8-6.4)	2.7 (0.8-9.4)
	Overweight (25–29.9 kg/m^2)	21.6	3.3	25.2	1.0	1.3 (0.8-2.0)	1.1 (0.6-1.8)	1.0 (0.6-1.8)
	Obese I (30–34.9 kg/m^2)	19.7	3.2	19.0	0.8	1.5 (0.9-2.5)	1.2 (0.7-2.2)	1.0 (0.6-1.9)
	Obese II (35–39.9 kg/m^2)	17.9	2.8	10.0	0.7	**2.6 (1.6-4.3)**	**2.3 (1.4-4.0)**	**1.8 (1.1-3.1)**
	Obese III (≥40.0 kg/m^2)	13.4	2.4	9.3	0.6	**2.1 (1.2-3.6)**	1.8 (1.0-3.3)	1.4 (0.8-2.5)
Waist circumference	High (>88 cm)	73.8	3.9	59.5	1.1	**1.9 (1.3-2.9)**	**1.7 (1.1-2.6)**	**2.1 (1.1-4.0)**
Blood pressure	High (≥120/80 mm Hg)	42.3	3.8	33.9	1.1	**1.4 (1.0-2.0)**	1.4 (1.0-2.0)	1.3 (0.8-1.9)
Serum HDL cholesterol	Low (<50 mg/dL)	44.2	4.5	36.3	1.1	1.4 (1.0-2.0)	1.2 (0.8-1.7)	0.8 (0.5-1.2)
Fasting LDL cholesterol	High (>100 mg/dL)	68.1	6.0	62.6	1.6	1.3 (0.7-2.3)	1.2 (0.7-2.2)	1.1 (0.6-2.0)
Fasting serum triglycerides	High (≥150 mg/dL)	33.1	5.2	17.8	1.2	**2.3 (1.4-3.7)**	**1.7 (1.0-2.9)**	1.3 (0.7-2.4)
Fasting plasma glucose	High (≥100 mg/dL)	36.8	5.1	27.2	1.4	1.6 (1.0-2.5)	**1.8 (1.0-3.1)**	1.6 (0.9-2.7)
Hemoglobin A1C	High (≥5.7%)	20.8	2.7	15.7	0.9	1.4 (1.0-2.0)	1.4 (1.0-2.1)	1.2 (0.7-1.9)
Metabolic syndrome	Meets 3 or more criteria	42.1	5.4	23.1	1.4	**2.4 (1.5-3.9)**	**2.3 (1.3-3.9)**	1.8 (1.0-3.2)
Hemoglobin	Low (<12 g/dL)	6.8	1.5	11.0	0.6	**0.6 (0.4-1.0)**	**0.6 (0.4-1.0)**	0.6 (0.4-1.1)
Hematocrit	Low (<36%)	10.3	2.1	12.6	0.7	0.9 (0.5-1.3)	0.7 (0.4-1.2)	0.9 (0.5-1.5)
RBC count	Low (<4.2 × 10^6 cells/μL)	25.9	3.7	20.5	1.0	1.4 (0.9-2.0)	1.4 (0.9-2.1)	**1.7 (1.1-2.6)**
Mean Cell Volume	Low (<80 fL)	7.1	1.7	8.1	0.5	0.9 (0.5-1.5)	0.9 (0.5-1.5)	0.9 (0.5-1.5)
	High (>100 fL)	3.9	1.8	0.9	0.2	**4.6 (1.6-13.2)**	2.1 (0.8-6.0)	2.5 (0.9-7.1)
Serum ferritin	Low (<15 μg/L)	12.6	3.1	17.3	0.8	0.7 (0.4-1.3)	0.7 (0.4-1.2)	0.8 (0.5-1.5)
	High (>150 μg/L)	10.7	3.1	6.6	0.7	1.6 (0.8-3.1)	1.4 (0.7-2.8)	1.2 (0.6-2.4)
Serum iron	Low (<40 μg/dL)	11.3	3.0	13.8	1.1	0.8 (0.4-1.7)	0.7 (0.4-1.2)	0.6 (0.3-1.1)
Serum transferrin receptor	High (>5.33 mg/L)	7.9	2.1	9.7	0.8	0.8 (0.4-1.5)	0.7 (0.4-1.4)	0.8 (0.5-1.5)
Serum transferrin saturation	Low (<15%)	25.4	4.4	26.7	1.5	0.9 (0.6-1.5)	0.8 (0.5-1.4)	0.8 (0.5-1.5)
Serum TIBC	High (>460 μg/dL)	7.2	2.9	6.8	0.9	1.1 (0.4-2.7)	1.0 (0.4-2.9)	1.3 (0.4-3.6)
Serum PLP (vitamin B6)	Low (<20 μmol/L)	25.2	5.1	14.1	1.4	**2.1 (1.3-3.4)**	1.6 (0.9-2.8)	1.2 (0.7-2.1)
Serum vitamin C	Low (<11.4 μmol/L)	18.5	6.1	7.0	0.9	**3.0 (1.3-6.8)**	2.0 (0.7-5.6)	1.3 (0.4-4.1)
Serum 25OH Vitamin D	Low (<30 nmol/L)	11.0	2.8	7.5	0.8	1.5 (0.9-2.6)	**2.0 (1.1-3.7)**	**2.1 (1.0-4.1)**

[a] Unadjusted, [b] Adjusted for survey cycle, age, race/ethnicity, education, marital status, income poverty ratio, employment, and health insurance, [c] Adjusted for model 2 covariates and BMI (except where BMI category is the outcome), alcohol use, smoking, history of arthritis, asthma, chronic bronchitis, cancer, diabetes, and thyroid conditions, and number of previous pregnancies.

Table S5. Cardiovascular, metabolic, hematologic, and micronutrient status measures among women with BMI < 30 by prescription opioid status, NHANES 1999–2018.

	**Rx Opioid Users**			**Unexposed Control**			**Model 1** [a]	**Model 2** [b]	**Model 3** [c]
**Measure**	*n*	**Mean**	**SE**	*n*	**Mean**	**SE**	*p*	*p*	*p*
BMI (kg/m^2)	195	24.2	0.3	4535	23.8	0.1	0.28	0.53	0.57
Waist circumference (cm)	194	85.1	1.0	4501	82.9	0.2	**0.02**	0.12	0.35
Systolic BP (mm Hg)	191	110.6	1.1	4426	109.1	0.2	0.18	0.39	0.22
Diastolic BP (mm Hg)	191	68.8	0.9	4426	67.5	0.2	0.15	0.42	0.31
HDL (mg/dL)	188	56.9	1.3	4361	60.2	0.3	**0.01**	0.08	0.43
Fasting serum LDL cholesterol (mg/dL)	86	109.8	4.0	1953	102.9	0.8	0.09	0.30	0.43
Fasting serum triglycerides (mg/dL)	87	119.7	11.3	1961	89.2	2.3	**0.009**	0.07	0.10
Fasting glucose (mg/dL)	88	92.6	1.2	1977	91.9	0.4	0.57	0.40	0.81
Hemoglobin A1C (%)	190	5.14	0.03	4384	5.15	0.01	0.57	0.35	0.42
Hemoglobin (g/dL)	191	13.56	0.09	4394	13.44	0.03	0.18	0.40	0.95
Hematocrit (%)	191	39.7	0.2	4394	39.5	0.1	0.37	0.51	0.85
RBC count (million cells/µL)	191	4.40	0.03	4394	4.43	0.01	0.25	0.28	0.17
Mean cell volume (fL)	191	90.6	0.5	4394	89.3	0.1	**0.006**	**0.01**	0.08
Serum ferritin (µg/L)	155	58.4	5.5	3413	50.0	1.1	0.13	0.13	0.19
Serum iron (µg/dL)	94	75.5	3.6	1941	89.4	1.1	**0.0003**	**0.003**	**0.004**
Serum transferrin receptor (mg/L)	116	3.59	0.33	2626	3.34	0.04	0.46	0.58	0.43
Serum transferrin saturation (%)	94	20.9	1.1	1938	25.3	0.3	**0.0002**	**0.002**	**0.002**
Serum total iron binding capacity (µg/dL)	94	371.3	9.5	1938	365.2	1.8	0.52	0.74	0.63
Serum folate (nmol/L)	189	38.6	1.4	4360	43.2	0.4	**0.003**	**0.01**	0.10
Serum vitamin B12 (pmol/L)	117	375.1	15.0	2493	424.9	35.1	0.20	0.32	0.46
Plasma homocysteine (µmol/L)	84	7.64	0.37	1558	6.94	0.08	0.06	0.14	0.14
Serum PLP (vitamin B6) (nmol/L)	69	80.8	15.3	1429	71.6	3.1	0.56	0.53	0.44
Serum retinol (µmol/L)	84	1.97	0.10	1543	1.88	0.02	0.37	0.51	0.60
Serum vitamin C (µmol/L)	56	50.6	3.8	1167	57.8	1.1	0.08	0.13	0.39
Serum 25OH vitamin D (nmol/L)	144	73.0	3.1	3174	69.7	0.7	0.30	0.70	0.69

[a] Unadjusted, [b] Adjusted for survey cycle, age, race/ethnicity, education, marital status, income poverty ratio, employment, and health insurance, [c] Adjusted for model 2 covariates and BMI (except where BMI is the outcome), alcohol use, smoking, history of arthritis, asthma, chronic bronchitis, cancer, and diabetes, and number of previous pregnancies.

Table S6. Health and nutrition status using defined using clinically relevant cutoffs among women with BMI <30 by prescription opioid use status.

		Rx Opioid		Control		Model 1 [a]	Model 2 [b]	Model 3 [c]
**Measure**	**Cutoff**	**%**	**SE**	**%**	**SE**	**OR (CI)**	**OR (CI)**	**OR (CI)**
Waist circumference	High (>88 cm)	38.3	4.0	28.9	0.8	**1.5 (1.1-2.1)**	1.3 (1.0-1.9)	1.1 (0.6-2.0)
Blood pressure	High (≥120/80 mm Hg)	21.7	3.0	16.6	0.6	1.4 (1.0-2.0)	1.3 (0.9-1.8)	1.3 (0.9-1.9)
Serum HDL cholesterol	Low (<50 mg/dL)	34.0	4.0	24.6	0.9	**1.6 (1.1-2.2)**	1.4 (1.0-1.9)	1.2 (0.8-1.7)
Fasting LDL cholesterol	High (>100 mg/dL)	56.1	5.7	48.9	1.4	1.3 (0.8-2.2)	1.2 (0.7-1.9)	1.2 (0.7-1.9)
Fasting serum triglycerides	High (≥150 mg/dL)	24.6	4.9	9.0	0.8	**3.3 (1.9-5.7)**	**3.0 (1.7-5.3)**	**2.6 (1.3-5.0)**
Fasting plasma glucose	High (≥100 mg/dL)	18.1	3.8	13.0	0.9	1.5 (0.9-2.5)	1.7 (0.9-3.1)	1.4 (0.7-2.8)
Hemoglobin A1C	High (≥5.7%)	7.2	2.2	4.3	0.3	1.7 (0.9-3.4)	1.9 (0.8-4.2)	1.9 (0.9-4.2)
Metabolic syndrome	Meets 3 or more criteria	23.8	5.4	6.5	0.6	**4.5 (2.5-8.0)**	**4.6 (2.4-8.9)**	**3.4 (1.4-8.3)**
Hemoglobin	Low (<12 g/dL)	4.5	1.5	7.6	0.4	0.6 (0.3-1.1)	0.5 (0.3-1.1)	0.6 (0.3-1.2)
Hematocrit	Low (<36%)	8.4	2.1	10.5	0.6	0.8 (0.5-1.3)	0.7 (0.4-1.3)	0.8 (0.5-1.5)
RBC count	Low (<4.2 × 10^6 cells/μL)	24.7	3.7	23.0	0.9	1.1 (0.7-1.6)	1.1 (0.7-1.6)	1.0 (0.7-1.6)
Mean Cell Volume	Low (<80 fL)	4.5	1.5	5.0	0.4	0.9 (0.5-1.9)	0.9 (0.5-1.8)	1.0 (0.5-2.0)
	High (>100 fL)	4.3	1.9	0.8	0.2	**5.6 (2.0-15.3)**	**3.0 (1.0-8.7)**	2.7 (0.9-7.9)
Serum ferritin	Low (<15 μg/L)	12.5	2.9	16.0	0.6	0.8 (0.5-1.3)	0.8 (0.5-1.3)	0.8 (0.5-1.4)
	High (>150 μg/L)	8.7	3.1	3.8	0.4	**2.3 (1.1-5.1)**	2.1 (0.9-4.6)	2.1 (1.0-4.5)
Serum iron	Low (<40 μg/dL)	16.1	4.4	9.5	0.7	1.9 (0.9-4.2)	1.6 (0.8-3.3)	1.6 (0.8-3.3)
Serum transferrin receptor	High (>5.33 mg/L)	6.5	2.0	6.5	0.5	1.0 (0.5-2.0)	0.9 (0.5-1.7)	1.0 (0.5-2.2)
Serum transferrin saturation	Low (<15%)	33.8	4.9	20.2	1.0	**2.0 (1.3-3.2)**	**1.8 (1.1-3.0)**	**1.8 (1.1-3.0)**
Serum TIBC	High (>460 μg/dL)	8.8	3.5	7.8	0.7	1.1 (0.5-2.7)	1.0 (0.4-2.5)	1.2 (0.5-2.8)
Serum PLP (vitamin B6)	Low (<20 μmol/L)	15.7	4.2	10.2	0.8	1.6 (0.9-3.3)	1.4 (0.7-3.0)	0.8 (0.3-2.0)
Serum vitamin C	Low (<11.4 μmol/L)	5.1	3.6	5.0	0.6	1.0 (0.2-4.3)	0.8 (0.2-3.2)	0.6 (0.1-3.9)
Serum 25OH Vitamin D	Low (<30 nmol/L)	5.7	2.1	4.9	0.5	1.2 (0.5-2.6)	1.5 (0.6-3.7)	1.7 (0.7-4.2)

[a] Unadjusted, [b] Adjusted for survey cycle, age, race/ethnicity, education, marital status, income poverty ratio, employment, and health insurance, [c] Adjusted for model 2 covariates and BMI, alcohol use, smoking, history of arthritis, asthma, chronic bronchitis, cancer, diabetes, and thyroid conditions, and number of previous pregnancies.

Table S7. Cardiovascular, metabolic, hematologic, and micronutrient status measures among women with BMI ≥ 30 by prescription opioid status, NHANES 1999–2018.

Measure	Rx Opioid Users			Unexposed Control			Model 1 [a]	Model 2 [b]	Model 3 [c]
	n	Mean	SE	*n*	Mean	SE	*p*	*p*	*p*
BMI (kg/m²)	209	38.0	0.5	2699	37.0	0.1	**0.03**	0.06	0.35
Waist circumference (cm)	203	113.9	1.2	2636	111.1	0.4	**0.03**	0.10	0.78
Systolic BP (mm Hg)	202	116.5	0.9	2624	116.8	0.3	0.74	0.51	0.21
Diastolic BP (mm Hg)	202	71.6	0.8	2624	71.8	0.2	0.79	0.25	0.14
HDL (mg/dL)	200	48.8	0.9	2595	49.0	0.3	0.84	0.46	0.08
Fasting serum LDL cholesterol (mg/dL)	90	115.1	3.9	1143	113.6	1.3	0.73	0.52	0.82
Fasting serum triglycerides (mg/dL)	91	148.1	12.6	1153	120.8	3.1	**0.03**	0.44	0.49
Fasting glucose (mg/dL)	92	102.4	2.4	1164	102.6	0.9	0.94	0.36	0.35
Hemoglobin A1C (%)	202	5.58	0.07	2616	5.50	0.02	0.28	0.41	0.99
Hemoglobin (g/dL)	202	13.36	0.11	2622	13.34	0.03	0.83	0.49	0.13
Hematocrit (%)	202	39.5	0.3	2622	39.5	0.1	0.89	0.58	0.14
RBC count (million cells/µL)	202	4.57	0.03	2622	4.57	0.01	0.87	0.83	0.57
Mean cell volume (fL)	202	86.7	0.5	2622	86.6	0.2	0.83	0.39	0.27
Serum ferritin (µg/L)	149	61.2	5.4	2029	60.5	1.5	0.89	0.67	0.21
Serum iron (µg/dL)	80	68.3	3.6	1124	69.2	1.4	0.81	0.83	0.99
Serum transferrin receptor (mg/L)	123	3.92	0.12	1624	3.83	0.05	0.48	0.12	0.07
Serum transferrin saturation (%)	80	19.7	1.3	1122	19.7	0.5	0.98	0.98	0.90
Serum total iron binding capacity (µg/dL)	80	358.9	8.3	1122	362.9	2.4	0.65	0.73	0.91
Serum folate (nmol/L)	201	34.2	1.6	2595	37.8	1.0	0.05	0.06	0.18
Serum vitamin B12 (pmol/L)	116	374.3	18.9	1368	370.7	7.2	0.86	0.92	0.47
Plasma homocysteine (µmol/L)	66	7.74	0.38	819	6.92	0.10	0.06	0.12	0.19
Serum PLP (vitamin B6) (nmol/L)	75	34.2	4.2	791	46.4	2.4	**0.02**	0.06	0.09
Serum retinol (µmol/L)	65	1.86	0.08	811	1.74	0.02	0.16	0.45	0.30
Serum vitamin C (µmol/L)	54	39.7	4.6	727	44.2	1.3	0.33	0.67	0.75
Serum 25OH vitamin D (nmol/L)	160	55.1	2.1	1751	54.4	0.9	0.72	0.44	0.83

[a] Unadjusted, [b] Adjusted for survey cycle, age, race/ethnicity, education, marital status, income poverty ratio, employment, and health insurance, [c] Adjusted for model 2 covariates and BMI (except where BMI is the outcome), alcohol use, smoking, history of arthritis, asthma, chronic bronchitis, cancer, and diabetes, and number of previous pregnancies.

Table S8. Health and nutrition status using defined using clinically relevant cutoffs among women with BMI ≥ 30 by prescription opioid use status.

		Rx Opioid		Control		Model 1 [a]	Model 2 [b]	Model 3 [c]
**Measure**	**Cutoff**	**%**	**SE**	**%**	**SE**	**OR (CI)**	**OR (CI)**	**OR (CI)**
Waist circumference	High (>88 cm)	98.9	0.6	99.3	0.2	Not analyzed due to near 100% prevalence		
Blood pressure	High (≥120/80 mm Hg)	39.3	3.7	40.2	1.2	1.0 (0.7-1.3)	0.9 (0.6-1.3)	0.8 (0.5-1.1)
Serum HDL cholesterol	Low (<50 mg/dL)	55.3	3.8	57.4	1.2	0.9 (0.7-1.3)	0.8 (0.6-1.1)	**0.6 (0.5-0.9)**
Fasting LDL cholesterol	High (>100 mg/dL)	62.3	6.1	64.5	2.0	0.9 (0.5-1.6)	0.7 (0.4-1.3)	0.7 (0.4-1.2)
Fasting serum triglycerides	High (≥150 mg/dL)	36.4	6.1	24.8	1.6	**1.7 (1.0-2.9)**	1.2 (0.7-2.0)	1.2 (0.7-2.0)
Fasting plasma glucose	High (≥100 mg/dL)	42.6	6.1	37.5	1.8	1.2 (0.7-2.1)	1.1 (0.6-1.9)	1.1 (0.6-1.9)
Hemoglobin A1C	High (≥5.7%)	21.8	3.3	23.2	1.0	0.9 (0.6-1.4)	0.9 (0.6-1.4)	0.7 (0.5-1.1)
Metabolic syndrome	Meets 3 or more criteria	49.3	6.2	39.8	1.6	1.5 (0.9-2.4)	1.1 (0.7-2.0)	1.1 (0.6-1.8)
Hemoglobin	Low (<12 g/dL)	10.2	2.2	10.5	0.7	1.0 (0.6-1.6)	1.1 (0.7-1.9)	1.2 (0.7-2.2)
Hematocrit	Low (<36%)	14.1	2.8	11.0	0.7	1.3 (0.9-2.1)	1.5 (0.9-2.6)	1.7 (1.0-2.9)
RBC count	Low (<4.2 × 10^6 cells/µL)	19.6	3.0	12.3	0.8	**1.7 (1.2-2.6)**	**1.6 (1.0-2.5)**	**1.8 (1.1-2.8)**
Mean Cell Volume	Low (<80 fL)	10.9	2.2	11.3	0.7	1.0 (0.6-1.5)	1.1 (0.7-1.8)	1.2 (0.8-1.9)
	High (>100 fL)	1.0	0.8	0.3	0.1	4.0 (0.7-21.9)	2.8 (0.5-15.4)	1.4 (0.3-7.0
Serum ferritin	Low (<15 µg/L)	12.9	3.1	13.9	0.9	0.9 (0.5-1.6)	1.0 (0.6-1.7)	1.2 (0.6-2.1)
	High (>150 µg/L)	8.3	2.8	6.4	0.6	1.3 (0.6-2.9)	1.1 (0.5-2.5)	0.9 (0.4-2.0)
Serum iron	Low (<40 µg/dL)	15.1	3.4	18.0	1.4	1.0 (0.5-1.8)	0.8 (0.5-1.5)	0.8 (0.4-1.4)
Serum transferrin receptor	High (>5.33 mg/L)	17.4	3.5	11.9	1.1	1.6 (1.0-2.6)	**1.9 (1.0-3.3)**	**2.2 (1.3-3.9)**
Serum transferrin saturation	Low (<15%)	37.3	6.1	35.3	1.8	1.1 (0.7-1.8)	1.2 (0.7-2.1)	1.2 (0.7-2.0)
Serum TIBC	High (>460 µg/dL)	9.6	3.3	5.7	0.9	1.8 (0.8-4.0)	2.2 (0.9-5.3)	2.2 (0.8-5.9)
Serum PLP (vitamin B6)	Low (<20 µmol/L)	31.8	5.3	20.7	1.9	**1.8 (1.1-3.0)**	1.5 (0.9-2.7)	1.3 (0.7-2.3)
Serum vitamin C	Low (<11.4 µmol/L)	17.0	5.5	8.7	1.5	**2.2 (1.1-4.3)**	1.6 (0.7-3.6)	1.2 (0.6-2.2)
Serum 25OH Vitamin D	Low (<30 nmol/L)	14.2	2.9	13.6	1.1	1.1 (0.7-1.7)	1.5 (0.9-2.4)	1.5 (0.9-2.6)

[a] Unadjusted, [b] Adjusted for survey cycle, age, race/ethnicity, education, marital status, income poverty ratio, employment, and health insurance, [c] Adjusted for model 2 covariates and BMI, alcohol use, smoking, history of arthritis, asthma, chronic bronchitis, cancer, diabetes, and thyroid conditions, and number of previous pregnancies.
